# The effect of an educational intervention to improve tuberculosis infection control among nurses in Ibadan, south-west Nigeria: a quasi-experimental study

**DOI:** 10.1186/s12912-020-00474-2

**Published:** 2020-08-28

**Authors:** Patrick Aboh Akande

**Affiliations:** 1 Department of Public Health, School of Health Care Sciences, Sefako Makgatho Health Sciences University, Pretoria, South Africa; 2 APIN Public Health Initiatives, Abuja, Nigeria

**Keywords:** Effect, Educational, Intervention, Tuberculosis, Infection, Control, Nurses

## Abstract

**Background:**

Nurses are particularly vulnerable to acquiring tuberculosis (TB) because they are in the frontline of patient care. There is inadequate implementation of cost-effective TB infection control (TBIC) measures in most health facilities. Training has been shown to be effective in improving the knowledge and work practices of nurses. This study sought to utilize a multi-method educational intervention to improve the TBIC-related knowledge and practices of nurses in two secondary health facilities in Ibadan, South-West Nigeria.

**Methods:**

This quasi-experimental study involved 200 nurses (100 each in the intervention and comparison groups). Baseline data were collected in May 2014. This was followed by training of the nurses in the intervention group. After 6 months, the second wave of data was collected and the nurses in the comparison group also received the training thereafter. The final wave of data collection took place 12 months after the commencement of the study. The mean scores of the nurses were determined and comparison was made between both groups at different time points using independent *t*-test.

**Results:**

The nurses in both groups were statistically comparable in their socio-demographic characteristics, and baseline mean knowledge (68.6 and 67.7%) and practice scores (79.1 and 80.6%) respectively. After the intervention group received the intervention, there were appreciable improvements in the scores at 6 months (knowledge – 85.9%, practice – 98.5%), which were significantly different from those of the comparison group (knowledge – 69.5%, practice – 78.8%). A large effect size was demonstrated in the improvement in knowledge score in the intervention group at 6 months compared with the other group (Cohen’s *d* = 1.7). Similarly, there were improvements in the scores of the nurses in the comparison group at 12 months after the group had also received the intervention (knowledge – 88.2%, practice – 93.5%). At this point, the mean scores between both groups were no longer significantly different.

**Conclusions:**

The improvement in post-intervention scores implies that the educational intervention adopted for this study was effective in improving TBIC among the nurses. It also underscores the importance of continuous training/retraining of nurses and other healthcare workers in improving and sustaining TBIC at health facilities.

## Background

Globally, tuberculosis (TB) is the leading cause of death due to an infectious disease [1]. It is caused by a microorganism called *Mycobacterium tuberculosis* (MTB) and commonly affects the lungs (pulmonary TB or PTB) and this accounts for about 85% of all TB cases [2]. TB can also affect other organs in the body (extrapulmonary TB): the lymph nodes, abdomen, bones and joints, pericardium, pleura, genitourinary system and meninges; and it can be generalized. PTB is the most important source of TB transmission as MTB is carried in air-borne droplets or aerosols produced when a person infected with PTB coughs, sneezes, spits, talks or sings. TB is preventable and there is affordable and effective treatment for it. In 2017, an estimated 10 million new cases of TB were recorded globally, with the African region accounting for 25% of these cases [1].

Nigeria is the most populous country in Africa, with a 2017 estimated population of about 182 million people [1]. It ranks sixth among the countries with the highest TB burden in the world and is included in the three lists compiled by World Health Organization (WHO) of 30 high burden countries (HBC) for (i) TB, (ii) TB/human immunodeficiency virus (TB/HIV), and (iii) multi-drug resistant TB (MDR-TB) for the period 2016–2020, with each list accounting for 85–89% of the global burden [3]. Nigeria is one of the 14 countries that appeared in all the three lists. Some of the countries that were in the WHO list of HBC for the year 2015 have made progress toward reducing their burden and have been removed from the lists for the 2016–2020 period while Nigeria has not made similar progress and is still present in the above lists. For instance, two countries (Afghanistan and Uganda) are no longer in the high burden countries while 14 countries have left the TB/HIV list. The MDR-TB list has similarly witnessed the exit of 6 countries [3]. Nigeria reported 418,000 incident TB cases in 2017, with an incidence rate of 219/100,000 population [1]. TB was successfully treated in 86% of all cases registered in the country in 2016; approximately 155,000 people died from it in 2017, with about 23% of these deaths (35,000) occurred in TB/HIV co-infected people. It has an MDR-TB/rifabutin-resistant (RR-TB) prevalence of 4.3 and 25% among new cases and previously treated cases respectively [1].

The spread of TB in health facilities, known as nosocomial TB transmission, poses a particular challenge for healthcare workers (HCWs) worldwide. The increased risk of nosocomial TB transmission among HCWs has been well-documented and the incidence of TB disease in HCWs is higher than in the general population [4–7]. This risk is worsened by the increased exposure of HCWs to infectious TB patients, especially when there is inadequate implementation of TB infection control (TBIC) measures [8–10]. Effective TBIC requires strict adherence to recommended control measures. Because of the diversity of the risk factors for TB transmission in health facilities, WHO has recommended the adoption of several TBIC measures [11]. These measures include (i) managerial measures; (ii) administrative measures – which are considered the first priority even in resource-limited settings;(iii) environmental control measures; and (iv) personal protective equipment (PPE) e.g. particulate respirator. Simple, practical and cost-effective interventions can be adopted in most low- and medium-income countries (LMIC) to reduce the exposure of HCWs to infectious TB patients [12]. These measures have been successfully implemented in most high-income countries and in some resource-limited settings [13]. Poor implementation of the recommended control measures by HCWs has however been reported [14–16]. The adoption and implementation of TBIC practices by HCWs are known to be positively influenced by good knowledge of occupational TB exposure [17, 18]. HCWs have been shown to have varying levels of knowledge and practices concerning TBIC and a good understanding of TBIC does not necessarily translate into adequate TBIC practices [19]. In addition to poor knowledge, weak managerial support, poor funding, limited work space and inadequate staffing have been identified as barriers to effective implementation of TBIC [20].

Surveys conducted in Nigeria to assess the level of implementation of TBIC practices in health facilities have generally shown poor results. At the time this study was undertaken, national guidelines for the implementation of TBIC had been rolled out. However, the policy had not fully trickled down to lower healthcare levels. In addition, training on the guidelines was yet to be widespread and administrative measures were still being put in place in most facilities providing care for TB patients [15, 20, 21]. Nursing staff are at high risk of acquiring TB because they are in the frontline of patient care and are frequently exposed to patients with infectious TB disease [22, 23]. They also play a critical role in curbing the spread of TB in health facilities. It is therefore imperative that they should be empowered with the necessary knowledge and skills to perform this function. The importance of training in enhancing work performance of nurses cannot be overemphasized. It ensures the acquisition of new knowledge and skills that can be effectively applied to work practices. Previous studies have noted post-training improvements in nurses’ knowledge and practices regarding general infection control [24–26]. A multi-method educational intervention involving the use of video presentation, group discussion, demonstration and handouts or lecture notes, deployed either as single entities or in combination, as an adjunct to the traditional method of didactic lectures has been proven to be more effective in improving knowledge and altering the professional practices of nurses [27, 28].

The aim of this study was to utilize a multi-method educational intervention to improve the TBIC-related knowledge and practices of nurses in two secondary health facilities in Ibadan, Nigeria. This will have implications for designing programmes to improve TBIC in health facilities.

## Methods

The study shares some similarities in the methods employed with an earlier one published by the author [29].

### Study design and setting

**Fig. 1:**
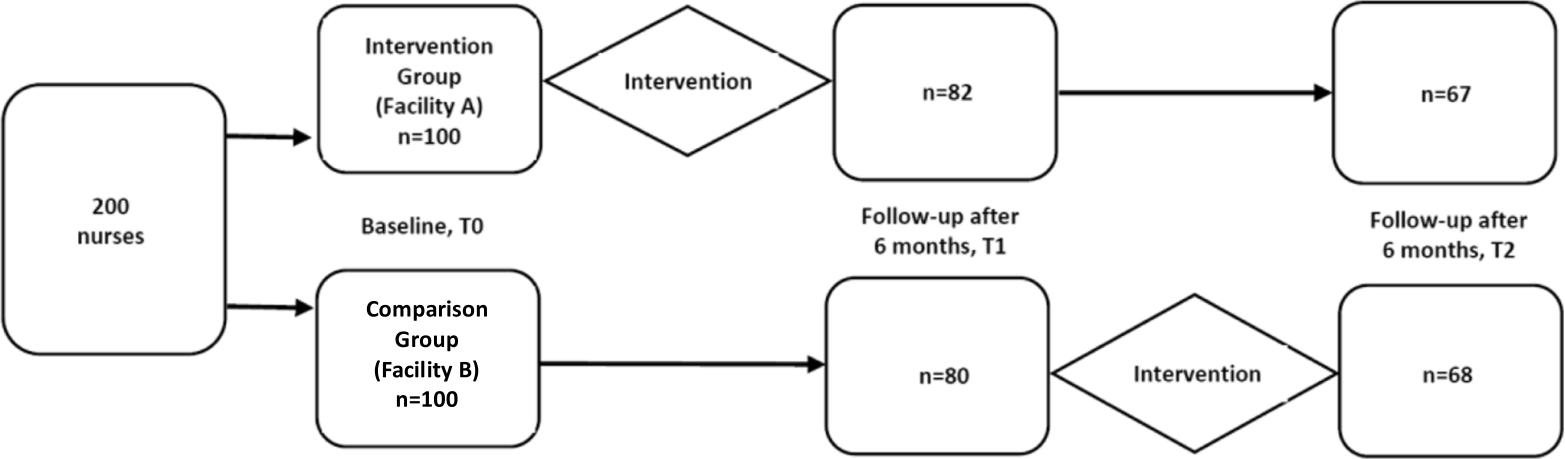
Flow diagram of the study

A quasi-experimental (pre- and post-test) design, with waitlist comparison group, was used for this study. A self-administered structured questionnaire was used to determine the TBIC-related knowledge and practices of nurses in an intervention group and a waitlist comparison group. It was administered to both groups at baseline (T0) after which the intervention group was exposed to the educational programme. The same cohorts of nurses in both groups were followed up and the questionnaire was again administered 6 months later (T1), as illustrated in Fig. 1. After the second data collection wave, the waitlist comparison group was also trained. Then 6 months later, final data collection was conducted (T2).

The specific objectives of the study were: (1) evaluate effectiveness of the training at 6 months, and (2) assess if there was sustained effect at 12 months.

The study was conducted in Ibadan, the capital city of Oyo State, South-West Nigeria. It is the third largest metropolitan area in Nigeria, and the largest by geographical area (3080 km^2^) with an estimated 2011 population of 3,034,206 (density of 985/km^2^) [30, 31]. Oyo State has the third highest TB burden in Nigeria, with 6901 cases reported in 2017 [32].

### Study population and sample

Nurses who work at two secondary health facilities in two separate Local Government Areas (LGAs), Ring Road State Hospital (Ibadan South-West LGA) and Adeoyo Maternity Teaching Hospital (Ibadan North LGA) constituted the study population: 173 and 217 nurses respectively (total = 390), from available administrative data at the study sites. The LGAs, which are non-contiguous, were purposively selected to avoid the effect of contamination. With an expected moderate effect size (ES > 0.50 <  0.80) in the TBIC knowledge of the nurses, a significance level of 5% and power of 80%, the study required at least 32 participants in each group [33]. However, because of the public health and health system considerations of the educational intervention, all available nurses at the study sites were encouraged to participate. Some of them could not be involved due to scheduling difficulties and since time was running out, a decision was taken to proceed to the next phase of the study after obtaining a reasonable sample size of 100 per site (total = 200).

### Educational intervention

The multi-method educational intervention, which took place over a 3-h period, consisted of didactic lectures in the form of Microsoft PowerPoint presentations prepared from WHO and U.S. Centers for Disease Control and Prevention (CDC) materials on TBIC; a 14-min video presentation entitled, *Implementing TB Infection Control in Outpatient Settings,* produced by CDC; as well as sessions for general discussion and practical demonstration [34–36]. A session on hand hygiene was included in the training as this has been recommended by WHO for implementation in the context of general infection control [11]. To serve as reminders, handouts or lectures notes were provided to the nurses at the end of the training session. Also, CDC-designed educational materials (signages, posters and stickers on TBIC workplace practices) were conspicuously displayed at the facility after the training. Adjustments in the training time was made to accommodate the nurses’ work schedule in order to train as many of them as possible: 5 training sessions were held for a group of 18–25 nurses each time, including evening sessions for the nurses that were on night shift. Generally, the educational materials covered the following TBIC-related topics: cause of TB, mode of transmission, symptoms and signs, infectiousness, risk factors and TBIC measures.

### Study instrument

The self-administered questionnaire employed for this study (Additional file 1) captured the socio-demographic characteristics of the nurses, as well as their TBIC-related knowledge and practices, utilizing scales adapted from an instrument previously used by Kanjee et al. to study TBIC in a high drug-resistance setting in South Africa [14]. The instrument was assessed for face validity by subject matter experts in Nigeria (two consultant chest physicians and two senior TB nurses) to ensure its relevance, adequacy and appropriateness while the content validity was improved upon by the review undertaken by the Sefako Makgatho Health Sciences University Research Ethics Committee. Based on the opinion of the experts, some of the initial instrument items were either deleted or rephrased. As shown by Cronbach’s alpha values of 0.6 and 0.8 respectively, both the knowledge and practice scales were found to have acceptable internal consistency. Pilot-testing of the instrument was conducted at one of the study sites on 15 nurses, who were eventually excluded from the main study. Their feedback on the clarity of the questions and challenges encountered in responding to them were also utilized to rephrase some of the items. The instrument was drafted in English and because this is the official language used by the participants and the medium of instruction during their training, there was no need for translation. The final knowledge scale had 33 items, with response options of “true” (score of “1”), “false” (score of “0”), or “I don’t know” (also scored as “0”). This scale had a maximum possible score of 33. Self-reported frequency of adherence to various TBIC practices was measured using six items, which were scored with a 5-point Likert-type scale: “never” (1 point), “rarely” (2), “sometimes” (3), “often” (4), and “always” (5). The participants could obtain a maximum possible score of 30 on this scale.

### Data collection

Data collection was carried out by two research assistants and a supervisor, who were trained for the purpose. The study participants received information about the study through their heads of units. On agreeing to participate in the study, each of the nurses received a copy of the information leaflet, consent form and study instrument. The self-administered questionnaire was then completed by the nurses and retrieved from them. Pre-test data collection took place in May 2014 (wave 1). Six months after the training was conducted on the intervention group, the questionnaire was again administered on both groups, in November 2014 (wave 2). After this, the waitlist comparison group received the intervention. At the end of another 6 months (in May 2015), the questionnaire was administered again on both groups (wave 3).

### Statistical analysis

The data collected were analyzed using SPSS Statistics version 24.0 (IBM Corp, Armonk, NY). Socio-demographic variables were described using means and frequencies while independent *t*-test and chi-square test (*χ*^2^) were utilized to compare continuous and categorical variables respectively, between the intervention and comparison groups. The knowledge and practice scores of the respondents at different time points were presented as mean percentage scores. Independent *t*-test was used to test for differences between the mean scores of both groups. Given the sample size change over time, an analysis of the baseline data of 6-month completers was also carried out (complete-case analysis). The level of statistical significance was set at *p* <  0.05. In addition, the scores were categorized into “good” and “poor” scores using cut-off points of 80 and 100% for knowledge and practices respectively. The cut-off for good practice score was fixed at 100% because optimal performance of TBIC measures is essential to minimize the nurses’ risk of contracting TB. The effect size (Cohen’s *d*) was calculated using the sixth-month measurement to investigate the magnitude of the change in the knowledge score of the intervention group resulting from the educational programme.

### Ethical considerations

Approval for the study was received from Sefako Makgatho Health Sciences University Research Ethics Committee (MREC/H/271/2013: PG), and Oyo State Ministry of Health Research Ethical Review Committee in Nigeria (AD 13/479/557). Oyo State Hospitals Management Board and the management of Adeoyo Maternity Teaching Hospital and Ring Road State Hospital, both in Ibadan, Oyo State, Nigeria, gave permission for the conduct of the study. Measures were taken to ensure the privacy and confidentiality of the participants, whose involvement was completely voluntary. Informed consent was obtained from each participant and their names, addresses and other unique identifiers were not included in the questionnaire, in order to ensure anonymity.

## Results

At baseline, completed questionnaires were collected from 100 nurses in each group (total = 200). During wave 2 data collection, the number of respondents dropped to 82 and 80 in the intervention and comparison groups respectively. These declined further to 67 (intervention group) and 68 (comparison group) at wave 3. Some of the nurses that dropped out of the study at months 6 and 12 complained of busy work schedules. Also, some of those in the intervention group claimed that there was no need to continue since they had already benefited from the training provided to the group after the baseline data collection, despite encouragement from the study team for them to complete the process.

### Socio-demographic characteristics of participants

Table 1 shows that the nurses in both facilities were comparable as there were no statistically significant differences in their socio-demographic characteristics at baseline.

**Table 1:** Socio-demographic characteristics of participants

	Intervention group (*N* = 100)	Comparison group (*N* = 100)	Test value	95% CI	*p*-value
*Continuous*					
*Variables*					
Age					
Mean (SD)	43.9 (8.88)	43.6 (9.11)	*t* = 0.28	− 2.15, 2.87	0 .78
Experience					
Mean (SD)	19.6 (9.66)	19.0 (9.81)	*t* = 0.44	−2.11, 3.33	0.66
*Categorical*					
*Variables*					
Sex, n(%)					
Female	96 (96)	98 (98)	*χ* ^2^ = 0.69		0.68*
Male	4 (4)	2 (2)			
Professional rank, n(%)					
Nursing Officer	18 (40)	21 (42)	*χ* ^2^ = 1.76		0.78
Senior Nursing Officer	22 (40)	21 (21)			
Principal Nursing Officer	22 (22)	19 (19)			
Assistant Chief Nursing Officer	5 (5)	9 (9)			
Chief Nursing Officer	33 (60)	30 (58)			
Marital status, n(%)					
Married	90 (90)	93 (93)	*χ* ^2^ = 0.58		0.45
Unmarried	10 (10)	7 (7)			

*Fisher’s *p*-value

### Scores of respondents

As illustrated in Table 2, the mean knowledge at baseline was not significantly different between the groups, *t* (198) = 0.62, *p* = 0.54. Similarly, their mean practice scores were not significantly different, *t* (198) = − 0.66, *p* = 0.51. After the training had been received by the intervention group, data collected at 6 months showed that there was an appreciable increase in the mean knowledge score in the intervention group while the comparison group only experienced a slight increase. The mean knowledge scores were statistically significantly different between both groups at this time, *t* (160) = 10.7, *p* <  0.001. Similarly, the mean practice score of the intervention group showed an improvement while there was a decline in that of the comparison group, and the difference in the scores between both groups was statistically significant, *t* (160) = 9.39, *p* <  0.001. After the training of the comparison group, measurement taken at 12 months revealed that the mean knowledge score of the comparison group improved from the 6 months value while there was a slight drop in that of the intervention group. The difference between the mean knowledge scores of both groups at 12 months was however not statistically significant, *t* (133) = − 1.85, *p* = 0.07. Likewise, the difference in the mean practice scores between both groups at the time was also not statistically different, *t* (133) = − 1.83, *p* = 0.07, although it improved in the comparison group from the 6 months value while the intervention group experienced a slight decrease.

**Table 2:** Mean scores of respondents at different time points

	Number of participants (N)		Mean score, % (SD)				
	Intervention group	Comparison group	Intervention group	Comparison group	*t*-value	95% CI	*p*-value
Baseline							
Knowledge	100	100	68.6 (9.83)	67.7 (10.9)	0.62	−1.99, 3.80	0.54
Practice	100	100	79.1 (15.1)	80.6 (15.5)	−0.66	−5.70, 2.83	0.51
6 months							
Knowledge	82	80	85.9 (9.26)	69.5 (10.3)	10.7	13.4, 19.5	< 0.001*
Practice	82	80	93.5 (8.32)	78.8 (11.4)	9.39	11.6, 17.8	< 0.001*
12 months							
Knowledge	67	68	84.8 (11.8)	88.2 (9.70)	−1.85	−7.10, 0.24	0.07
Practice	67	68	90.7 (10.3)	93.5 (7.85)	−1.83	−6.00, 0.24	0.07
6-month completers							
Baseline							
Knowledge	82	80	68.8 (10.2)	66.8 (10.8)	1.23	−1.23, 5.29	0.22
Practice	82	80	79.8 (14.1)	81.4 (14.7)	−0.74	−6.12, 2.80	0.46

*p < 0.05

An analysis of the baseline scores of the 6-month completers (those that that were still retained in the study at 6 months) revealed the same result as that of all respondents at baseline – no significant difference between both groups.

### Score categories of respondents

Table 3 shows that at baseline, using cut-off scores of 80% (knowledge) and 100% (practice) to categorize the scores of the nurses, the majority of them in both groups had poor scores on both scales. At 6 months, the proportions in the intervention group (which had earlier received the training) that recorded good scores on both scales had increased. These proportions were higher than those with good scores in the comparison group. After the comparison group had also benefitted from the training, there was an increase in the proportions in this group with good knowledge and practices scores from the 6 months values while the proportions dropped in the intervention group.

**Table 3:** Score categories of respondents at different time points

	Baseline, n (%)		6 months, n (%)		12 months, n (%)	
	Good	Poor	Good	Poor	Good	Poor
Knowledge						
Intervention group	12 (12.0)	88 (88.0)	59 (72.0)	23 (28.0)	43 (64.2)	24 (35.8)
Comparison group	9 (9.0)	91 (91.0)	10 (12.5)	70 (87.5)	53 (77.9)	15 (22.1)
Practice						
Intervention group	4 (4.0)	96 (96.0)	44 (53.7)	38 (46.3)	22 (32.8)	45 (67.2)
Comparison group	8 (8.0)	92 (92.0)	6 (7.50)	74 (92.5)	36 (52.9)	32 (47.1)

### Effect size determination at 6 months

The effect size for the knowledge scores resulting from the intervention received by nurses in the intervention group compared with those in the comparison group was calculated at 6 months using

$$ \mathrm{Cohen}'\mathrm{s}\ d={\mathrm{Mean}}_1-{\mathrm{Mean}}_2/\mathrm{Pooled}\ \mathrm{Standard}\ \mathrm{Deviation}=\left(85.9-69.5\right)/9.8=1.7. $$

This is a large effect size, according to Cohen’s classification [33].

## Discussion

This quasi-experimental study was conducted to determine the effect of a multi-method educational intervention aimed at improving TBIC among nurses at two secondary health facilities in Ibadan, Oyo State, Nigeria. The nurses in both groups were comparable in their socio-demographic characteristics and their mean knowledge and practice scores at baseline. Using the cut-off points of 80 and 100% for good knowledge and practice scores respectively, the majority of nurses in both groups also had poor scores on the TBIC-related knowledge and practice scales at baseline.

The poor levels of baseline TBIC knowledge and practice found in this study are consistent with reports from other studies in Nigeria, where generally, poor levels concerning TBIC have been demonstrated among HCWs [16, 19, 20]. This also agrees with reports from other countries such as Russia and Georgia [17, 37]. On the contrary, “good” or “adequate” TBIC knowledge among HCWs have been reported by some other investigators, although lower cut-off points were used for these studies. For instance, using a lower cut-off (70%) than that used in the present study to categorize good knowledge, Bhebhe et al. observed that 89.2% of HCWs in their study in Lesotho had “appropriate” TBIC knowledge [8]. Even the mean score of 61.5% reported by them was lower than 68.6 and 67.7% observed in the present study. Similarly, 69% of HCWs in the study by Buregyeya et al. were said to have adequate TBIC knowledge, based on a lower cut-off of 70% [38]. Several studies from LMIC are in support of the results of the current study regarding the baseline TBIC practices. Researchers have reported inadequate implementation of TBIC measures in Nigeria, South Africa, Lesotho, Ethiopia, among others [9, 10, 15, 39]. In contrast, an overall “good” TBIC practice was reported by Temesgen and Demissie in Ethiopia, using a lower cut-off point of 50%, though implementation of “specific practices” was noted to be poor [40]. It is noteworthy that TBIC guidelines had just been released in Nigeria at the time of the present study and implementation was still in its early stages [15, 21]. The finding of high proportions of nurses with poor levels of TBIC knowledge and practices at baseline in this study is therefore not unexpected.

After the training of the nurses in the intervention group, there was considerable improvement in the mean scores for knowledge and practices at 6 months, as well as in the proportions that had good scores on the two scales. Their counterparts in the comparison group, who had not received the training at this time, only had a slight improvement in their mean knowledge score while there was a small drop in their mean practice score. The differences in the mean scores between nurses in the two facilities were statistically significant on the two scales at 6 months after the intervention group had been trained. These findings suggest that the educational intervention had indeed contributed to improving their knowledge and practices, considering that the scores were statistically similar between both groups at baseline. Furthermore, a large effect size of 1.7 on the knowledge scale (measured at 6 months) resulted from the training received by nurses in the intervention group. This outcome concurs with reports by earlier investigators who noted improvements in knowledge and practices among nurses in Nigeria and elsewhere after an educational intervention [24–26, 41, 42]. After the intervention was implemented in the comparison group, there were similar improvements in the mean scores of this group at 12 months on both knowledge and practice scores. In the intervention group, there were slight reductions in the mean scores at 12 months. The increase in the mean scores of the comparison group on both scales was enough to ensure that the scores were not statistically significantly different from those of the intervention group at 12 months. This indicates that the implementation of the intervention activities in the comparison group had resulted in the group catching up with the intervention group. The post-intervention increase in the mean scores of both groups observed in the present study (6 months for the intervention group and 12 months for the comparison group) is in alignment with the findings in a study conducted by Buregyeya et al. in Uganda, where most of the HCWs had correct TBIC knowledge, beliefs and practices after national TB guidelines had been introduced and training on TBIC had taken place in the few years preceding the study [38]. Although information on the pre-training levels was not provided, the investigators reported that those that did not receive the training had poor knowledge and practices.

At 12 months, the nurses in the intervention group experienced a slight decline in both mean knowledge and practice scores. This is consistent with findings from similar follow-up studies involving nurses where their scores decreased slightly in follow-up (wave 3) measurement after appreciable improvement was recorded in the immediate post-intervention measurement (wave 2) [25, 26]. This, however, contrasts with a report by Price which showed good retention of the positive improvement 12 months later [43]. The decline in scores at 12 months in the present study suggests a loss of knowledge and a tendency to revert to old practices with the passage of time and emphasizes the need for retraining and continuous professional education for nurses as this is necessary to reinforce important TBIC messages and the practice of infection prevention skills [44].

The outcomes of this study show that the educational intervention, which had the added benefit of incorporating video presentation, group discussion and demonstration methods into the traditional lecture method, was effective in improving the knowledge and practice scores of the nurses. Several researchers have demonstrated the impact of adopting these methods in various combinations to complement the lecture method instead of the use of lecture method alone. In a study that examined the combination of lecture notes and structured group discussion (intervention group) versus lecture notes alone (control group), Johnson et al. reported that the mean scores of the intervention group in a surgical nursing course was significantly higher than that of the control group [27]. Lee et al., in an investigation of the effect of an instructional video on paediatric intraosseous needle insertion, observed that there was improved clinical skills learning outcomes in the intervention group compared with the traditional face-to-face didactic teaching method (control group) [28]. On the contrary, students’ performance outcomes remained unchanged when training conducted with video method (intervention) was compared with the combination of lecture and demonstration methods (control) in an evaluation conducted by Kelly et al. concerning the use of instructional videos to equip student nurses with clinical skills [45]. The researchers recommended that instructional videos should be used to complement the lecture method rather than replace it. A blend of several methods was used in the present study as this has been proven to be effective [27, 28]. The discussion method is thought to provide the learners with the opportunity to express and discuss their concerns and clarify concepts within the group while the video method is related to increased cognitive and procedural knowledge, and high learner satisfaction with the teaching experience [27, 46].

### Limitation of the study

The nurses in the study reported their TBIC practices using self-administered questionnaires. Direct observation of the practices could not be carried out due to time constraint and the cost of engaging several research personnel as observers. Self-reports tend to be exaggerated by respondents (social desirability bias), as previously observed in the study by Engelbrecht et al. [16].

## Conclusions

This study revealed that the multi-method educational intervention was effective in improving the nurses’ post-intervention TBIC knowledge and practices. This approach, which incorporates didactic lectures, video presentation, group discussion and demonstration, as well as the use of TBIC educational materials, is recommended for TBIC-related training and retraining of nurses and other HCWs, as part of continuous professional development. This would enhance the acquisition and retention of knowledge and skills that are necessary to minimize the risk of contracting TB by nurses and other HCWs, and improve TBIC in health facilities.

## Supplementary information

**Additional file 1.** Study Questionnaire.

## Data Availability

The datasets used and analyzed during the current study are available from the corresponding author on reasonable request.

## Abbreviations

CDC: U.S. Centers for Disease Control and Prevention
CI: Confidence interval
HBC: High burden countries
HCW: Healthcare worker
HIV: Human Immunodeficiency Virus
LGA: Local Government Area
LMIC: Low- and medium-income countries
MDR-TB: Multi-drug resistant tuberculosis
MTB: *Mycobacterium tuberculosis*
PPE: Personal protective equipment
PTB: Pulmonary tuberculosis
RR-TB: Rifampicin-resistant tuberculosis
TB: Tuberculosis
TBIC: Tuberculosis infection control
WHO: World Health Organization

## References

[1] World Health Organization (2018). Global tuberculosis report 2018.

[2] Cruz-Knight W, Blake-Gumbs L (2013). Tuberculosis: an overview. Prim Care.

[3] World Health Organization (2015). Use of high burden country lists for TB by WHO in the post-2015 era.

[4] Joshi R, Reingold AL, Menzies D, Pai M. Tuberculosis among health care workers in low- and middle-income countries: a systematic review. PLoS Med. 2006;3(12):e494. 10.1371/journal.pmed.0030494. 10.1371/journal.pmed.0030494 PMC1716189 17194191

[5] Menzies D, Joshi R, Pai M (2007). Risk of tuberculosis infection and disease associated with work in health care settings. Int J Tuberc Lung Dis.

[6] Malangu N, Legothoane A (2013). Analysis of occupational infections among health care workers in Limpopo province of South Africa. Global J Health Sci.

[7] Claassens MM, van Schalkwyk C, du Toit E, Roest E, Lombard CJ, Enarson DA, Beyers N, Borgdorff MV. Tuberculosis in healthcare workers and infection control measures at primary healthcare facilities in South Africa. PLoS One. 2013;8(10):e76272. 10.1371/journal.pone.0076272. 10.1371/journal.pone.0076272 PMC3788748 24098461

[8] Bhebhe LT, Van Rooyen C, Steinberg WJ. Attitudes, knowledge and practices of healthcare workers regarding occupational exposure of pulmonary tuberculosis. Afr J Prim Healthcare Fam Med. 2014;6(1):E1–6. 10.4102/phcfm.v6i1.597. 10.4102/phcfm.v6i1.597 PMC4502877 26245412

[9] Malangu N, Mngomezulu M (2015). Evaluation of tuberculosis infection control measures implemented at primary health care facilities in Kwazulu-Natal province of South Africa. BMC Infect Dis.

[10] Mugomeri E, Chatanga P, Lefunyane M, Ruhanya V, Nyandoro G, Chin’ombe N (2015). Adherence to tuberculosis infection control guidelines by nurses in Lesotho. Am J Infect Control.

[11] World Health Organization (2009). Policy on TB infection control in health care facilities, congregate settings and households.

[12] World Health Organization (2019). WHO guidelines on tuberculosis infection prevention and control 2019 update.

[13] Jensen PA, Lambert LA, Iademarco MF, Ridzon R (2005). Centers for Disease Control and Prevention. Guidelines for preventing the transmission of mycobacterium tuberculosis in health care settings. MMW Recomm Rep.

[14] Kanjee Z, Amico KR, Li F, Mbolekwa K, Moll AP, Friedland GH (2012). Tuberculosis infection control in a high drug-resistance setting in rural South Africa: information, motivation, and behavioural skills. J Infect Public Health.

[15] Ogbonnaya LU, Chukwu JN, Uwakwe KA, Oyibo PG, Ndukwe CD (2011). The status of tuberculosis infection control measures in health care facilities rendering joint TB/HIV services in "German leprosy and tuberculosis relief association" supported states in Nigeria. Niger J Clin Pract.

[16] Engelbrecht M, van Rensburg AJ, Kigozi G, van Rensburg HCJ (2016). Factors associated with good TB infection control practices among primary healthcare workers in the Free State Province, South Africa. BMC Infect Dis.

[17] Woith WM, Volchenkov G, Larson JL (2010). Russian health care workers’ knowledge of tuberculosis and infection control. Int J Tuberc Lung Dis.

[18] Kanjee Z, Catterick K, Moll AP, Amico KR, Friedland GH (2011). Tuberculosis infection control in rural South Africa: survey of knowledge, attitude and practice in hospital staff. J Hosp Infect.

[19] Kuyinu YA, Mohammed AS, Adeyeye OO, Odugbemi BA, Goodman OO, Odusanya OO (2016). Tuberculosis infection control measures in health care facilities offering TB services in Ikeja local government area, Lagos, South West, Nigeria. BMC Infect Dis.

[20] Akosu TJ, Tolulope A, Agbo HA (2015). Assessment of tuberculosis infection control measures in primary and secondary health care facilities in Enugu. IOSR J Dent Med Sci.

[21] Federal Ministry of Health (2008). National Tuberculosis and Leprosy Control Programme (NTBLCP). The national guidelines for TB infection control.

[22] Chanda D, Gosnell D (2006). The impact of tuberculosis on Zambia and the Zambian nursing workforce. Online J Issues Nurs.

[23] Christopher DJ, James P, Daley P, Armstrong L, Isaac BTJ, et al. High annual risk of tuberculosis infection among nursing students in South India: a cohort study. PLoS One. 2011;6(10):e26199. 10.1371/journal.pone.0026199. 10.1371/journal.pone.0026199 PMC3192164 22022565

[24] Galal YS, Labib JR, Walaa A, Abouelhamd WA (2014). Impact of an infection-control program on nurses’ knowledge and attitude in pediatric intensive care units at Cairo University hospitals. J Egypt Public Health Assoc.

[25] Adly RM, Amin FM, Abd El Aziz MA (2014). Improving nurses' compliance with standard precautions of infection control in pediatric critical care units. World J Nurs Sci.

[26] Suchitra JB, Lakshmi DN (2007). Impact of education on knowledge, attitudes and practices among various categories of health care workers on nosocomial infections. Ind J Med Micribiol.

[27] Johnson JP, Mighten A (2005). A comparison of teaching strategies: lecture notes combined with structured group discussion versus lecture only. J Nurs Edu.

[28] Lee JC, Boyd R, Stuart P (2007). Randomized controlled trial of an instructional DVD for clinical skills teaching. Emerg Med Australas.

[29] Akande PA. Knowledge and practices regarding tuberculosis infection control among nurses in Ibadan, south-west Nigeria: a cross-sectional study. BMC Health Serv Res. 2020;20:280. 10.1186/s12913-020-05156-y. 10.1186/s12913-020-05156-y PMC7132981 32252759

[30] Aremu FJ, Olugbire OO, Adebayo DA, Apata OV (2015). Socio-economic characteristics of Bodija sawn wood market in Ibadan, Oyo state, Nigeria. J Soc Sci Public Policy.

[31] National Bureau of Statistics (2012). Annual abstract of statistics.

[32] Federal Ministry of Health (2017). National Tuberculosis and Leprosy Control Programme (NTBLCP). Annual Report.

[33] Cohen J (1988). Statistical power analysis for the behavioral sciences.

[34] World Health Organization (2008). Participant's manual for IMAI TB infection control training at health facilities.

[35] World Health Organization (WHO) (2006). Centers for Disease Control and Prevention (CDC). Tuberculosis infection control in the era of expanding HIV care and treatment: an addendum to WHO guidelines for the prevention of tuberculosis in health care facilities in resource-limited settings.

[36] Centers for Disease Control and Prevention (2012). Implementing TB infection control in outpatient settings.

[37] Mirtskhulava V, Whitaker JA, Kipiani M, Harris DA, Tabagari N, Owen-Smith AA, Kempker RR, Blumberg HM (2015). Determinants of tuberculosis infection control-related behaviors among healthcare workers in the country of Georgia. Infect Control Hosp Epidemiol.

[38] Buregyeya E, Kasasa S, Mitchell EMH (2016). Tuberculosis infection control knowledge and attitudes among health workers in Uganda: a cross-sectional study. BMC Infect Dis.

[39] Tamir K, Wasie B, Azage M (2016). Tuberculosis infection control practices and associated factors among health care workers in health centers of west Gojjam zone, Northwest Ethiopia: a cross-sectional study. BMC Health Serv Res.

[40] Temesgen C, Demissie M (2014). Knowledge and practice of tuberculosis infection control among health professionals in Northwest Ethiopia; 2011. BMC Health Serv Res.

[41] Mockiene V, Suominen T, Välimäki M, Razbadauskas A (2010). Impact of intervention programs on nurses’ knowledge, attitudes, and willingness to take care of patients with human immunodeficiency virus/acquired immunodeficiency syndrome: a descriptive review. Medicina (Kaunas).

[42] Odutayo PO, Olaogun AA, Oluwatosin AO, Ogunfowokan AA (2013). Impact of an educational program on the use of standardized nursing languages for nursing documentation among public health nurses in Nigeria. Int J Nurs Knowl.

[43] Price SL (2009). Becoming a nurse: a meta-study of early professional socialization and career choice in nursing. J Adv Nurs.

[44] Bluestone J, Johnson P, Fullerton J, Carr C, Alderman J, BonTempo J (2013). Effective in-service training design and delivery: evidence from an integrative literature review. Hum Resour Health.

[45] Kelly M, Lyng C, McGrath M, Cannon G (2009). A multi-method study to determine the effectiveness of, and student attitudes to, online instructional videos for teaching clinical nursing skills. Nurse Educ Today.

[46] Cardoso AF, Moreli L, Braga FTMM, Vasques CI, Santos CB, Carvalho EC (2012). Effect of a video on developing skills in undergraduate nursing students for the management of totally implantable central venous access ports. Nurse Educ Today.

