# Supplementary material for: The effect of an educational intervention to improve tuberculosis infection control among nurses in Ibadan, south-west Nigeria: a quasi-experimental study
Source: BMC Nurs. 2020 Aug 28;19:81. doi: 10.1186/s12912-020-00474-2 (PMC7456062; doi:10.1186/s12912-020-00474-2)
Supplement: Supplementary file 1 — Additional file 1. Study Questionnaire. [file 12912_2020_474_MOESM1_ESM.docx]

**Additional File 1: Study Questionnaire**

**The effect of an educational intervention to improve tuberculosis infection control among nurses in Ibadan, south-west Nigeria: a quasi-experimental study**

## Section A

Please circle the features that apply to you and fill in the required information where indicated.

1. Sex: You are:
   1. Male
   2. Female
2. How old are you? (years)
3. What is your marital status?
   1. Single
   2. Married
   3. Divorced
   4. Widowed
4. What is your professional rank? (Circle only one.)
   1. Nursing Officer
   2. Senior Nursing Officer
   3. Principal Nursing Officer
   4. Assistant Chief Nursing Officer
   5. Chief Nursing Officer
5. How long have you been a nurse? (years)

## Section B

For each of the following symptoms, please tick (√) “true” if you consider it a symptom of TB in the lungs, “false” if it is not, or “I don’t know”) if you are not sure. Please tick only one option for each symptom.

| **SN** | **Symptom** | **True** | **False** | **I don’t know** |
| --- | --- | --- | --- | --- |
| 1 | Blurry vision (False) |  |  |  |
| 2 | Coughing for longer than 2-3 weeks (True) |  |  |  |
| 3 | Coughing up blood (True) |  |  |  |
| 4 | Ear pain (False) |  |  |  |
| 5 | Fever (True) |  |  |  |
| 6 | Memory loss (False) |  |  |  |
| 7 | Night sweats (True) |  |  |  |
| 8 | Pain with urination (False) |  |  |  |
| 9 | Watery eyes (False) |  |  |  |
| 10 | Weight loss (True) |  |  |  |

The following are statements about the mode of spread and risk of TB. For each statement, please tick (√) “true” if the statement is true, “false” if it is not, or “I don’t know” if you are not sure. Please tick only one option for each statement.

| **SN** | **Statement** | **True** | **False** | **I don’t know** |
| --- | --- | --- | --- | --- |
| 11 | TB can be spread to others through semen or vaginal fluid (False) |  |  |  |
| 12 | TB can be spread to others through the air (True) |  |  |  |
| 13 | TB can be spread to others through contact with blood (False) |  |  |  |
| 14 | Patients with TB disease can infect other people by coughing (True) |  |  |  |
| 15 | Patients with TB disease can infect other people by sharing food (False) |  |  |  |
| 16 | Patients with TB disease can infect other people by talking or  singing (True) |  |  |  |
| 17 | Patients with TB disease can infect other people by sneezing (True) |  |  |  |
| 18 | Patients with TB disease are more likely to infect others if they  cough up a lot of sputum (True) |  |  |  |
| 19 | Treating TB patient with the right drugs does not affect how  infectious they are (False) |  |  |  |
| 20 | Healthcare workers in the outpatient clinic have the same risk of  getting TB as any other person (False) |  |  |  |
| 21 | An HIV-positive person has the same risk of getting TB as an  HIV-negative person (False) |  |  |  |
| 22 | An HIV-positive staff member cannot get sick with TB if they  practise TB infection control measures (False) |  |  |  |
| 23 | It is alright for HIV-positive staff who are healthy to work in TB  High-risk areas of the hospital (False) |  |  |  |

The following are statements about practising TB infection control measures. For each statement, please tick (√) “true” if the statement is true, “false” if it is not, or “I don’t know”) if you are not sure. Please tick only one option for each statement.

| **SN** | **Statement** | **True** | **False** | **I don’t know** |
| --- | --- | --- | --- | --- |
| 24 | When entering the outpatient clinic, every patient should be asked if they are coughing (True) |  |  |  |
| 25 | Patients who are identified as presumptive TB cases should not be separated from other patients in the waiting area as this will be seen to be discriminating against them (False) |  |  |  |
| 26 | A coughing/sneezing patient should be instructed to cover their  mouth with a handkerchief, tissue or their arm while coughing/ sneezing (True) |  |  |  |
| 27 | If coughing/sneezing TB patients or presumptive cases use handkerchief or tissue to cover their mouth while coughing or sneezing, that is usually enough to protect the health care worker (False) |  |  |  |
| 28 | If a coughing patient has not been diagnosed as a case of TB, it is  not necessary to instruct them to cover their mouth while coughing (False) |  |  |  |
| 29 | A coughing patient should be instructed to collect a sputum sample in the clinic toilet (False) |  |  |  |
| 30 | Opening windows in a room with a coughing patient has no effect on the spread of TB (False) |  |  |  |
| 31 | If a fan is used in a room, opening windows will not provide  additional benefit for TB infection control (False) |  |  |  |
| 32 | The windows in a room where there is a TB patient should not be  opened because they have to be hidden from other people (False) |  |  |  |
| 33 | Presumptive TB cases in the waiting area should wait just as long as everyone else, and should not be rushed through the queue (False) |  |  |  |

## Section C

The following are statements about practising TB infection control measures. For each statement, please tick (√) the frequency with which you practise each of the measures. Please tick only one option for each statement.

| **SN** | **Task** | **Never** | **Rarely** | **Sometimes** | **Often** | **Always** |
| --- | --- | --- | --- | --- | --- | --- |
| 1 | How frequently do you ask each patient when they enter the clinic if they are coughing? |  |  |  |  |  |
| 2 | How frequently do you move coughing patients to wait at a nearby but separate waiting area? |  |  |  |  |  |
| 3 | How frequently do you instruct coughing patients to cover their mouth with tissues, handkerchiefs or their arm when coughing? |  |  |  |  |  |
| 4 | How frequently do you ensure collection of sputum samples from a patient is done outdoors or in separate, well-ventilated areas? |  |  |  |  |  |
| 5 | How frequently do you rapidly move coughing patient to the front of the queue so he/she is seen quickly to minimize the amount of time they spend in the clinic? |  |  |  |  |  |
| 6 | How frequently do you open windows in patient waiting area or a room where coughing patients are attended to (or check to see if they are open already)? |  |  |  |  |  |
